# Assessment of knowledge and perceptions of human papillomavirus vaccine and its determinants among women who have eligible daughters in Debre Berhan City, Ethiopia: a cross-sectional study

**DOI:** 10.3389/fonc.2024.1348288

**Published:** 2024-03-18

**Authors:** Abate Wondesen Tsige, Kassahun Dires Ayenew, Siraye Genzeb Ayele

**Affiliations:** ^1^ Department of Pharmacy, Clinical Pharmacy Unit, Debre Berhan University, Debre Berhan, Ethiopia; ^2^ Department of Midwifery, College of Health Sciences, School of Nursing and Midwifery, Addis Ababa University, Addis Ababa, Ethiopia

**Keywords:** cervical cancer, knowledge, perceptions, women, human papilloma virus, human papilloma virus vaccine

## Abstract

**Introduction:**

Globally, cervical cancer(CC) is the second most commonly diagnosed cancer and the fourth leading cause of cancer-related deaths in women. Human papillomavirus (HPV) infection is the leading cause of CC. Persistent infection with HPV accounts for 90% of all CC cases. The human papillomavirus vaccine has the great potential to prevent HPV-related infections for millions of women and men. The current study aimed to assess knowledge and perceptions towards the HPV vaccine and its determinants among women who have eligible daughters in Debre Berhan City, Ethiopia.

**Methods:**

A cross-sectional study was conducted from April 2, 2023, to May 15, 2023. A multistage sampling procedure was used to recruit 607 women participants. Descriptive statistics were used to summarize socio-demographic data. Univariable and multivariable binary logistic regression analyses were performed to measure the associations between the dependent and independent variables. A p-value of <0.05 was considered statistically significant.

**Results:**

More than three-fourths of the participants, 479 individuals (80%) were currently married, and 243(40.1%) had a diploma or higher education level. Of 456(75.12) participants reported, they had information about cervical cancer. For 449(73.9%) of the participants, television was the main evidence. The majority of 352(59.99%) participants knew the HPV vaccine could be offered to a female child aged 9-14 years old. Only 215(35.4%) participants think the HPV vaccine was safe and effective. Women who had a degree and above educational level were about 9 times more likely to have good knowledge about the HPV vaccine than study participants who did not read and write (AOR=9.21; 95% CI=2.82-12.16; p=0.004). Women who did not have information about the HPV vaccine before this study were about 80% less likely to have a positive perception of the HPV vaccine than participants who had earlier information about the HPV vaccine (AOR=0.8; 95%CI=0.63-0.49; P=003).

**Conclusion:**

Women had poor knowledge and perceptions about the HPV vaccine. Maternal marital status, age, and having information about the HPV vaccine were the only predictors of women’s knowledge of the HPV vaccine.

## Background

Cervical cancer(CC) is a malignant epithelial tumor that arises from normal cervical epithelium through the progressive development of low-grade and high-grade cervical intraepithelial lesions (1–3). Cervical cancer is the second most commonly diagnosed cancer and the fourth leading cause of cancer-related deaths in women worldwide. Above 560,000 new cases and about 275,000 deaths are recorded each year, with more than 80% occurring in developing countries. It is the most public gynecological cancer among women in sub-Saharan Africa (4). It is estimated that every year, around 3,235 deaths occur in Ethiopia due to cervical cancer disease (5).

In high-income countries, the incidence and mortality of CC have decreased by more than half over the past 30 years since formal screening programs and HPV vaccination programs were introduced (6). In developing countries, CC is a major problem due to limited awareness among the population, healthcare providers, and policy-makers (7).

Human papillomavirus infection is the leading cause of CC worldwide (8). Above 140 HPV genotypes have been identified and they are classified into high-risk (HR), probable high-risk (PHR), and low-risk (LR) types (9, 10). Almost 50 of these genotypes are identified to be oncogenic or HR types, which cause CC. Out of these, 15 HR-HPV genotypes: HPV-16, -18, -31, - 33, -35, -39, -45, -51, -52,-56, -58, -59, -68, - 73, and -82 cause more than 95% of all cases of CC (9). It could be prevented with safe sexual practices and the use of vaccines (11–13).

The human papillomavirus vaccine has the great potential to prevent HPV-related infections for millions of women and men worldwide (14). The HPV vaccine is safe, well-tolerated, and used to significantly reduce the incidence of HPV-associated precancerous lesions (10). World Health Organization(WHO) recommended that all countries introduce HPV vaccination for the primary prevention of CC, prioritizing the primary target group of young adolescent girls aged 9 to 14 years, before sexual exposure. As long as females have not already been infected with HPV, the vaccine is very successful (15, 16).

Ethiopia also launched the HPV vaccine for the first time with the provision of the Global Alliance for Vaccine and Immunization (GAVI) in 2018. Currently, the HPV vaccine is delivered to all 14-year-old girls through a school-based approach (12).

A cross-sectional study done in Malaysia among parents who have primary school students indicated that 62% of parents had poor knowledge about the HPV vaccine (17). Similarly, in a study done in Indonesia among mothers who have girls aged 12-15 years, only 44% of mothers had good knowledge (18) about the HPV vaccine.

There is a limited study evaluating the knowledge and perceptions towards human papillomavirus vaccine in Ethiopia. Therefore, the objective of this study was to assess knowledge and perceptions towards the human papillomavirus vaccine and its determinants among women who have eligible daughters at Debre Berhan City. The findings of the study will help to identify the level of knowledge and perceptions of women. In addition, a study was suggested focusing on mothers to improve the awareness of the HPV vaccine thus it is contributing to reducing the incidence of HPV infections and CC since mothers are directly faced with CC.

## Methods

### Study design, area, and period

A community-based cross-sectional study was done in Debre Berhan City from April 2, 2023, to May 15, 2023. Debre Berhan is found 130 km away from Addis Ababa, the capital city of Ethiopia The city has 10 administrative kebeles. According to the Debre Berhan City Administration, the total population in 2022/2023 was 188,513. There were three hospitals (two governmental and one private), three health centers, nine health posts, 10 private clinics, and 25 community pharmacies in the city that provided health services to the general population.

### Population

All mothers who had eligible daughters living in Debre Berhan city were the source population whereas mothers who had eligible daughters in the selected kebeles were the study population.

### Eligibility criteria

In the selected kebeles, all women who have eligible daughters(14 years to 18 years old) and who go to schools were eligible in the current study. Women who refused to participate in the current study were not eligible.

### Sample size determination

Using a single proportion formula, the sample size was calculated by the following assumptions: there was a study in Ethiopia, so, we used 56% prevalence (19) of women’s perceptions toward HPV vaccination at α= 0.05 significant level, 95% confidence interval, and margin of error (d) 5%.


$$\text{n}={\left(\text{Zα}/2\right)}^{2}\text{P}(1-\text{P})/{\text{d}}^{2}={\left(1.96\right)}^{2}0.56(1-0.56)/{\left(0.05\right)}^{2}=379$$

By considering the design effect of 1.5 and 10% non-respondents, a total of 607 women were recruited as study participants.

### Sampling procedures

**Figure 1:**
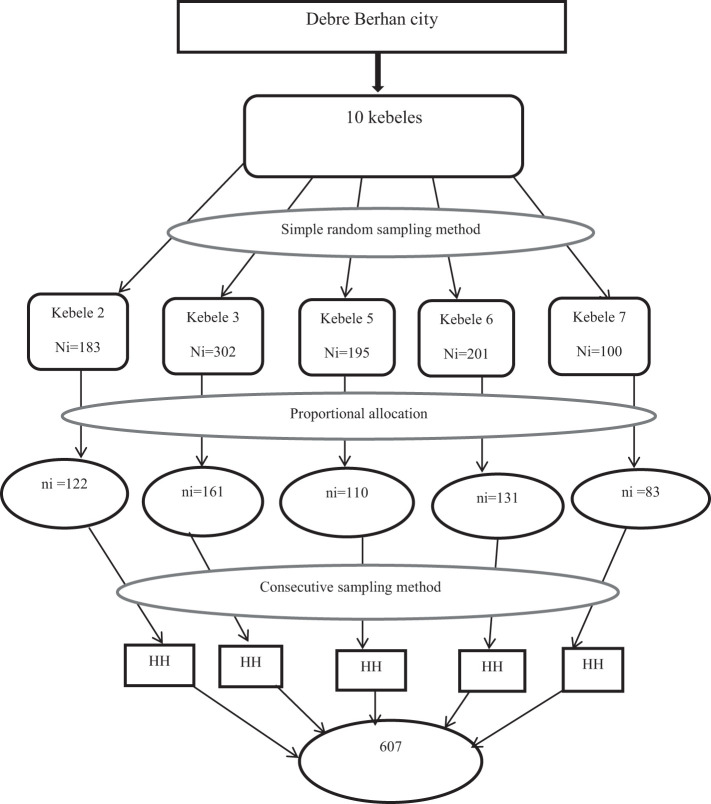
Schematic presentation of the sampling procedure for assessment of knowledge and perceptions about HPV vaccine in Debre Berhan city, 2023. HH, household.

A multi-stage sampling technique was applied to select kebeles. Five kebeles were selected from 10 kebeles using a simple random sampling method (lottery method). The final study participants were selected from each selected kebeles using the proportional allocation method. A list of households was taken from the registration book of each selected kebeles. Kebeles administrative office was used as the starting point for the selection of the first household (**Figure 1**).

### Variables

Women’s knowledge and perceptions of the HPV vaccine were the dependent variables whereas socio-demographic factors (age, religion, marital status, occupation, education status, number of children, number of daughters, and economic status) were the independent variables.

### Data collection technique

A pre-test was done in 5% (20) of study participants in kebele 10 before the actual data collection in the main study. The two-day training was given to data collectors and supervisors. There were three diploma nurses involved in the data collection and one BSc degree in public health involved in the supervision of the data collection process. The questionnaires used to collect data in the current study are found in **Supplementary Material** (**Supplementary File S1**).

Women’s knowledge was assessed using 13-item knowledge questions. Giving 0 for participants who didn’t correctly answer the questions and 1 for participants who correctly answered the questions. Then women’s knowledge was categorized as poor knowledge (0–7 out of 13 items), and good knowledge (8-13 out of 13 items) (21). Its Cronbach’s Alpha value was 0.66. Women’s perception was also assessed using 12-item perception questions. Giving 0 for participants who had negative perceptions about the question and 1 for those who had positive perceptions about the question, then categorized as negative perception (0–6 out of 12 items), and positive perception (7-12 out of 12 items) (21). Its Cronbach’s Alpha value was 0.69.

### Data processing and analysis

Epi data version 4.2.0. and SPSS version 25 were used for data entry and analysis, respectively. Descriptive statistics, univariable, and multivariate binary logistic regression were used. A p-value <0.05 was considered statistically significant at 95% CI.

### Ethical consideration

From Asrate Weldeyes Health Sciences Campus of Debre Berhan University, Institutional Review Board (IRB) ethical clearance(IRB/03/28/2023) of the study was obtained. Written informed consent or voluntary participation was obtained from each participant. In addition, confidentiality of the information was assured by using code numbers rather than personal identification names.

## Results

### Demographic features of participants

More than three-fourths of the participants, 479 individuals (80%), were currently married, 243(40.1%) had a diploma or higher educational level. Out of the maternal professions represented, only 364 individuals (62.6%) had different professions, while the remaining 231 individuals (37.4%) were housewives. In the majority of participants, 238(39.2%) monthly incomes were below 2000, and 197(32.45%) of the participant’s husband’s educational level were degree and above. Additionally, the majority of participants 536(88.32%) had less than or equal to four children and 553(91.1% had one daughter aged 9-14 years in the house (**Table 1**).

**Table 1:** Demographic features of participants in Debre Berhan city, 2023 (N=607).

Variables		Number (Percent)
Age	19-30 31-40 41-50 ≥ 51	71(10.4) 269(43.8) 176(30) 91(15.8)
Marital status	Married Divorced Widowed	479(80) 76(13) 52 (7)
Level of mothers’ education	Cannot write and read Primary or secondary school Diploma and above	160(26.5) 204(33.4) 243(40.1)
Maternal occupation	Governmental Self-employed Housewife Others	198(35) 165(25.4) 231(37.4) 13(2.2)
Maternal monthly income	Below 2000 2001-5000 5001-8000 7801-10900 Above 10900	238(39.2) 157(25.9) 166(27.35) 38(6.26) 8(1.29)
Total children in the household	<=4 5 and above	536(88.32) 71(11.68)
Total daughters aged 9-14 years	One More than one	553(91.1) 54(8.9)

### Sources of information about the HPV vaccine

**Figure 2:**
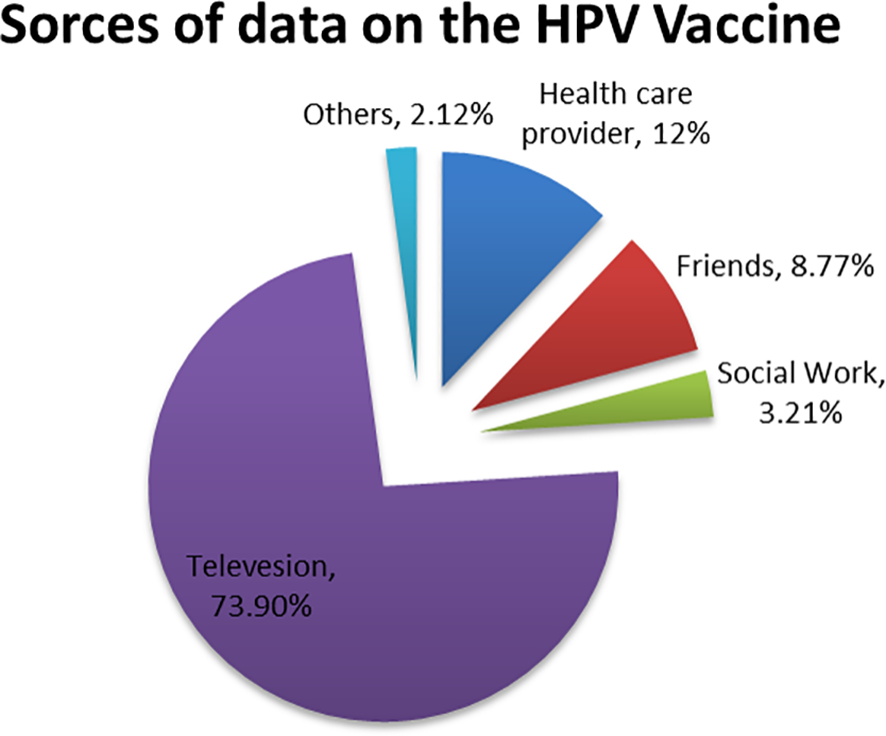
Sources of information for women about cervical cancer at Debre Berhan City, 2023.

For 449(73.9%) of the participants, television was the main evidence, followed by healthcare provider 73 (12%) and friends 53 (8.77%) (**Figure 2**).

### Women’s knowledge about HPV vaccine

**Figure 3:**
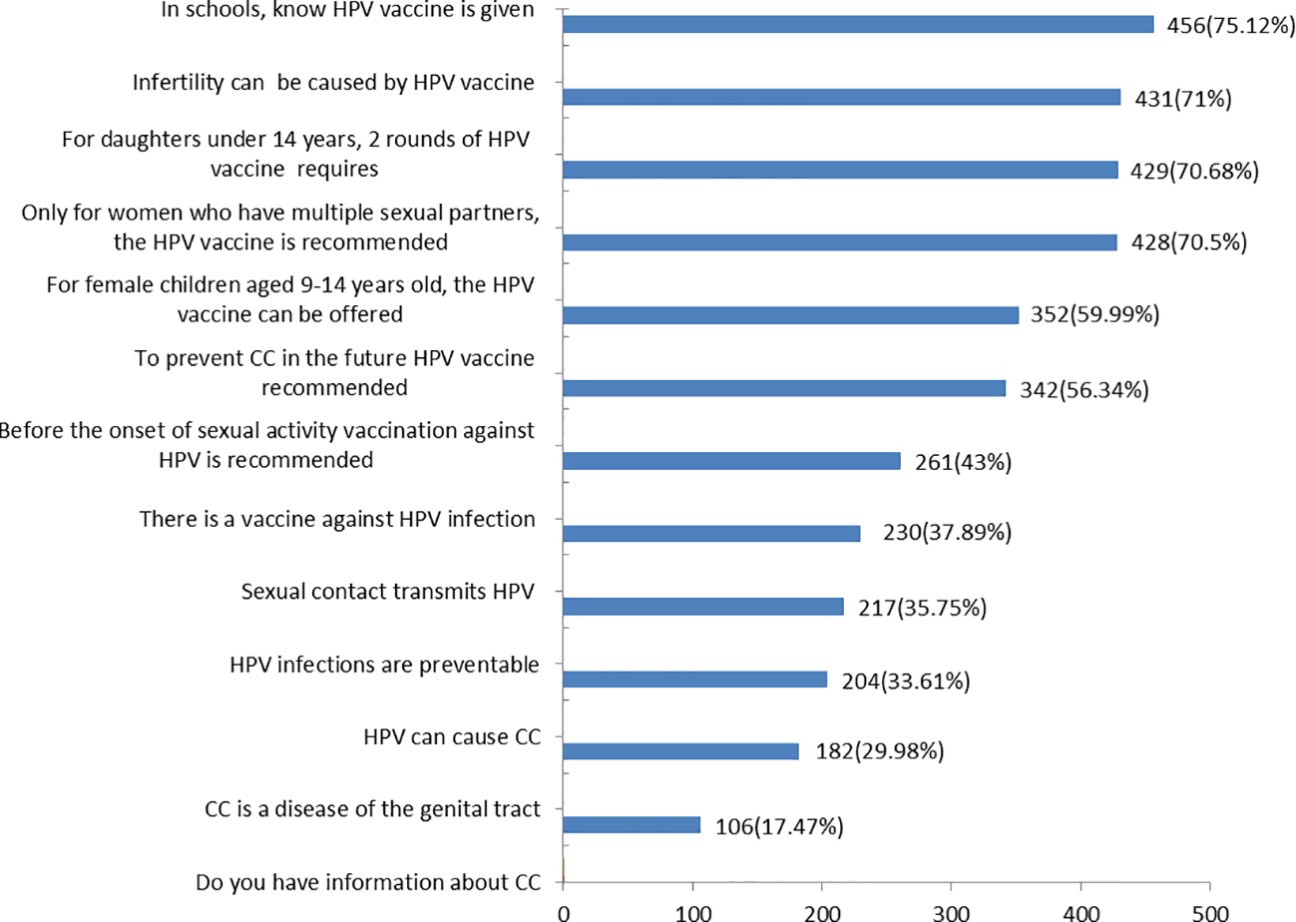
Correct responses of HPV vaccination knowledge items at Debre Berhan city, 2023 (n = 607).

Three-fourths of 456(75.12) of participants had information about CC. The majority 342(56.34%) of participants knew the existence of a vaccine against HPV infection, but only 182(29.98%) participants knew HPV can cause CC. Nearly two-thirds of 352(59.99%) of the participants knew the HPV vaccine could be offered to female children aged 9-14 years old (**Figure 3**).

### Women’s perception of the HPV vaccine

Above one-third 215(35.4%) of participants think the HPV vaccine was safe and effective, and 364(60%) study participants reported, they didn’t think their children are susceptible to HPV infection. The majority 491(80.89%) of women said that information on HPV helps them to decide whether their children should be vaccinated against HPV. Nearly two-thirds of 365(60.13%) of the participants reported being afraid of the mild side effects of the HPV vaccine for their daughter (**Table 2**).

**Table 2:** Women’s perception towards HPV vaccine in Debre Berhan City, 2023 (n=607).

Perception Item questions	Response	
	Yes	No
Do you think your daughter is susceptible to HPV infection	243(40)	364(60)
Do you think the HPV vaccine is safe and effective	215(35.4)	392(64.6)
Do you think being vaccinated for HPV reduces the risk of having an HPV infection	315(51.89)	292(48.11)
Do you think the HPV vaccine will not lead to complicated sexual activities	342(56.34)	265(43.66)
Do you think vaccinating your daughter against HPV will not encourage them to start sexual activity	317(52.22)	290(47.78)
Do you think the HPV vaccine promotes risky sexual behaviors among teenagers	176(29)	431(71)
Would you like to vaccinate your daughter against HPV if the vaccination is freely available?	397(65.41)	210(34.59)
Do you think to decide whether your children should be vaccinated against HPV, information on HPV helps you?	491(80.89)	116(19.11)
Are you afraid of mild side effects of the HPV vaccine for your daughter	365(60.13)	242(39.87)
Do you fear infertility from the HPV vaccine for your daughter in the future	217(35.75)	390(64.25)
Do you think the HPV vaccine is effective in preventing cervical cancer	363(59.8)	244(40.2)
Do you think only those who are promiscuous would benefit from the vaccine	153(25.21)	454(74.79)

### Predictors associated with women’s knowledge

Using binary logistic regression analysis, the univariable analysis found that eight variables were candidates for multivariable analysis. All of these variables were categorical, with five of them being multi-categorical maternal age, education, occupation, income, and marital status. The remaining variables were binary. To further investigate the association between these variables, a multivariable binary regression analysis was conducted with eight variables with the dependent variable. Finally, three variables were identified as significant associations between women’s knowledge of the HPV vaccine (**Table 3**).

Women within the 19-30 age groups were 4.6 times more likely to know about the HPV vaccine than women who were equal to or greater than 51 years old (AOR=4.6; 95% CI=0.23,0.78; p= 0.003).

Women who did not have information about the HPV vaccine before the current study were about 72% less likely to know about the HPV vaccine than women who had earlier information about the HPV vaccine (AOR=0.72; 95%CI=0.82,0.93; P=0.001) (**Table 3**).

**Table 3:** Predictors associated with women’s knowledge about HPV vaccine at Debre Berhan City, 2023 (n = 607).

Variables	Category	COR(95% CI)	AOR(95% CI)
Age	19-30 31-40 41-50 ≥ 51	3.62(2.53,5.48)* 2.88(0.58,2.61)* 3.11(2.24,3.43)* 1	4.6(0.23,0.78) ** 0.57(0.61,1.88) 3.14(0.41,3.73) 1
Marital status	Married Divorced Widowed	0.27(0.27,0.72)* 0.59(0.63,1.43) 1	4(1.4,2.58) ** 0.72(0.65,1.36) 1
Level of maternal education	Unable to read and write Read and write only Primary or Secondary school Diploma degree and above	1 3.27(2.13, 3.96)* 3.87(2.15, 8.85)* 21.80(10.90, 31.32)*	1 2.39(0.42, 4.74) 3.47(0.34, 6.50) 9.21(0.82, 12.16)
Maternal occupation	Governmental Self-employed Housewife Others	1 0.176(0.056,0.552)* 0.210(0.131,0.335)* 0.106(0.21,0.542)*	1 0.335(0.043,2.596) 1.596(0.497,5.124) 6.269(0.606,64.860)
Total children in the household	Less than or equal to 4 5 and above	1 0.90(0.64,0.70)*	1 4.25(3.17,4.40)
Total daughters aged 9-14 years	One More than one	1 2.52(0.60,3.91)	
Having information about the HPV vaccine	Yes No	1 0.27(0.85,0.89)*	1 0.72(0.82,0.93)**

**significance at p<0.05; COR, crude odds ratio; AOR, adjusted odds ratio; CI, confidence interval. * Variables candidate for a multivariable binary regression analysis.

### Predictors associated with women’s perception

Using binary logistic regression analysis, the univariable analysis found that seven variables were candidates for multivariable analysis. A multivariable binary regression analysis was conducted with seven variables with the dependent variable. Finally, information about the HPV vaccine was identified as a significant association between women’s perceptions towards the HPV vaccine (**Table 4**).

**Table 4:** Predictors associated with women’s perception of HPV vaccine at Debre Berhan City, 2023 (n = 607).

Variables	Category	COR(95% CI)	AOR(95% CI)
Age	19-30 31-40 41-50 ≥ 51	1 0.61(0.17,0.80)* 0.55(0.52,2.23)* 0.50(0.15,0.93)*	1 0.83(0.99,2.75) 0.22(0.30, 2.53) 0.66(0.17,3.44)
Marital status	Married Divorced Widowed	1 2.20(0.40,3.14) 3.75(0.33,3.17)	1 1.20(0.50,2.14) 2.15(0.43,2.22)
Level of maternal education	Unable to read and write Read and write only Primary or Secondary school Diploma and above	1 2.40(0.34,3.24) 3.33(0.77,4.26)* 2.60(0.34,2.70)*	1 0.24(0.27,3.73) 2.74(0.47,2.54) 1.72(0.51,3.80)
Maternal occupation	Governmental Self-employed Housewife Others	1 0.83(0.92, 0.70)* 0.30(0.13,0.81)* 2.83(0.76,8.80)	1 0.71(0.14,4.58) 2.15(0.54,7.88) 11.53(0.73,17.22)
Maternal monthly income	Below 2000 2001-5000 5001-8000 7801-10900 Above 10900	1 1.27(0.61,3.76) 2.52(0.50,3.27)* 3.49(2.58,3.66)* 1.38(0.57,4.23)*	1 0.51(0.38,1.56) 1.65(0.79,3.12) 1.66(0.69,5.11) 1.76(0.26,4.59)
No_ of daughters aged 9-14 years	One More than one	1 0.53(0.68,0.67)*	1 0.67(0.84,1.31)
Having information about the HPV vaccine	Yes No	1 0.56(0.72,0.82)*	1 0.80(0.63,0.49)**

**significance at p<0.05. * Variables candidate for a multivariable binary regression analysis.

Women who did not have information about the HPV vaccine before the current study were about 80% less likely to have a positive perception towards the HPV vaccine than participants who had earlier information about the HPV vaccine(AOR=0.8; 95%CI=0.63-0.49; P=003) (**Table 4**).

## Discussion

This current study found that study participants had low levels of knowledge and perceptions about the HPV vaccine. Which was comparable with studies conducted elsewhere (1, 15, 16, 21). This finding was in line with the study done in Debre Markos Town, Ethiopia (22) and Western Kenya (23).

Three-fourths of women (75.12%) had information about the HPV vaccine. This finding was similar to a study done in Brazil (75.91%) (24). However, our findings were greater than the studies in Bangladesh (56%) (25), Nigeria (36.5%) (15), Arab Communities (26.1%) (26), Kazakhstani(52%) (27), and Lebanon(34%) (28). The variation could be differences in the study setting, local cultural factors, and demographic, and clinical factors of study participants. Despite the development of evidence-based prevention strategies for CC in resource-limited settings such as Ethiopia, implementation and service uptake still need improvement (29).

In the current study, the study participants(74%) main source of information about the HPV vaccine was television. This finding was in line with a study done in the United Arab Emirates (8), but the study in Bangladesh (30%) (25) reported that the main source was newspapers while the study in Serbia (29.1%) (30) their main source of information was health care providers. These differences might be due to differences in the health-related information dissemination mechanism of each country’s policies and culture.

Nearly 30% of study participants knew HPV can cause CC. This finding was lower than the reported rate in Malaysia (86.8%) (17), Serbia (67.7%) (30), India(67.5%) (31), Indonesia (64.74%) (20), and Lebanon (34%) (28). This variation may be related to the participant’s level of educational status, culture, and religion. Cervical cancer is predominantly caused by persistent HPV infections (3). It can be due to HPV-related (early onset of sexual activity, multiple sexual partners, high-risk sexual partner, and early age at first birth history of vulvar or vaginal squamous intraepithelial neoplasia and immunosuppression) (32–34) and Non-HPV-related (low socioeconomic status, oral contraceptive use, cigarette smoking, and genetics) (35–37).

Around 38% of respondents knew the recommendation of the HPV vaccine before the onset of sexual activity. Our result was parallel to a study in Serbia (43.3%) (30), but studies done in Ghana (21.7%) (38) and Indonesia (24%) (20) found lower results. Routine human HPV vaccination for girls and boys at the target age 11–12 years (but can be given as early as 9 years) as part of the American College of Obstetricians and Gynecologists recommended adolescent vaccination platform (39). After the initiation of HPV 16 and 18 vaccination, HPV infections decreased by 83% in girls aged 15–19 years and by 66% in women aged 20–24 years (up to 8 years) (40).

The majority (60.13%) of participants reported being afraid of HPV vaccine side effects. Our finding was greatly higher than a study in Serbia (1.3%) (30). One of the arguments of the anti-vaccine movement that negatively impacted rates of coverage is the suspected side effects of the vaccine and vaccination against HPV is likely to decrease CC screening behaviors (24). The 9-valent HPV vaccine (Gardasil 9), the quadrivalent HPV vaccine (Gardasil), and the bivalent HPV vaccine (Cervarix) underwent several years of rigorous safety testing before being approved by the Food and Drug Administration (FDA) (41). Clinical trials involving over 15,000 women and men for Gardasil 9, over 30,000 for Cervarix, and over 29,000 for Gardasil have demonstrated their safety (42). Recent studies have revealed that more parents are expressing concerns about the safety of HPV vaccines, despite over 15 years of consistent evidence demonstrating their effectiveness and safety. A study from 2015 to 2018 showed a decline in the acceptance rate of HPV vaccines for children, from 13% to 23%. It is important to note that, like any vaccine or medicine, HPV vaccines can cause side effects such as pain, redness, swelling, dizziness, syncope (fainting), nausea, and headache (43).

### Strengths and limitations of the study

A large sample size is used to get a representative sample. Interviewer-administered questionnaires were used to avoid misunderstanding of the questions. A true cause-effect relationship between dependent and independent variables is not shown since this study used a cross-sectional design.

## Conclusions

Women had poor knowledge and perceptions about the HPV vaccine. Having information about the HPV vaccine was the only identified factor associated with women’s perception of the HPV vaccine.

To improve the knowledge and perceptions of mothers about their daughters’ HPV vaccine, the Ethiopian Ministry of Health should strengthen public knowledge and perceptions about HPV vaccine effectiveness and safety using health extension workers and mass media programs. Further research is also required with large study participants in this issue.

## Data availability statement

The original contributions presented in the study are included in the article/**Supplementary Material**. Further inquiries can be directed to the corresponding author.

## Ethics statement

The studies involving humans were approved by From Asrate Weldeyes Health Sciences Campus of Debre Berhan University, Institutional Review Board (IRB) ethical clearance(IRB/03/28/2023) of the study was obtained. The studies were conducted in accordance with the local legislation and institutional requirements. The participants provided their written informed consent to participate in this study.

## Author contributions

AT: Conceptualization, Data curation, Formal analysis, Funding acquisition, Investigation, Methodology, Project administration, Resources, Software, Supervision, Validation, Visualization, Writing – original draft, Writing – review & editing. KA: Conceptualization, Formal analysis, Software, Supervision, Validation, Visualization, Writing – original draft, Writing – review & editing. SA: Conceptualization, Data curation, Formal analysis, Funding acquisition, Investigation, Methodology, Project administration, Resources, Software, Supervision, Validation, Visualization, Writing – original draft, Writing – review & editing.
