# Supplementary material for: Assessment of knowledge and perceptions of human papillomavirus vaccine and its determinants among women who have eligible daughters in Debre Berhan City, Ethiopia: a cross-sectional study
Source: Front Oncol. 2024 Mar 18;14:1348288. doi: 10.3389/fonc.2024.1348288 (PMC10982310; doi:10.3389/fonc.2024.1348288)
Supplement: Supplementary file 1 [file DataSheet_1.docx]

**Part I: Socio-demographic Characteristics**

1. Age _____
2. Marital status__________
3. Level of maternal education________________
4. Maternal occupation __________________
5. Maternal monthly income______________________
6. Total children in the household­­­­­­­­____________
7. No_ of daughters aged 9-14 years______________________

**Part II: Having information about the HPV vaccine**

1. Have you ever heard about HPV vaccine before this study? 1. Yes 2. No
2. Source of information about HPV vaccine?

1. Friend 2.Television 3. internet 4. health care providers 5. brochure 6. Slogan 7. Others……

**Part III: Knowledge about HPV vaccine**

| Do you have information about cervical cancer? | 1. Yes 2. No |
| --- | --- |
| cervical cancer is a disease of the genital tract? | 1. Yes 2. No 3. I do not know |
| HPV can cause cervical cancer? | 1. Yes 2. No 3. I don’t know |
| HPV infections are preventable? | 1. Yes 2. No 3. I don’t know |
| Sexual contact transmits HPV? | 1. Yes 2. No 3. I do not know |
| There is a vaccine against HPV infection ? | 1. Yes 2. No 3. I don’t know |
| Before the onset of sexual activity vaccination against HPV is recommended? | 1. Yes 2. No 3. I don’t know |
| To prevent cervical cancer in the future HPV vaccine recommended? | 1. Yes 2. No 3. I don’t know |
| For female children aged 9-14 years old, the HPV vaccine can be offered? | 1. Yes 2. No 3. I don’t know |
| Only for women who have multiple sexual partners, the HPV vaccine is recommended? | 1. Yes 2. No 3. I don’t know |
| For daughters under 14 years, 2 rounds of HPV vaccine requires ? | 1. Yes 2. No 3. I don’t know |
| Infertility can be caused by HPV vaccine ? | 1. Yes 2. No 3. I don’t know |
| In schools, know HPV vaccine is given ? | 1. Yes 2. No 3. I don’t know |

**Part IV: Perception towards HPV vaccine**

| Do you think your daughter is susceptible to HPV infection? | 1. Yes 2. No |
| --- | --- |
| Do you think the HPV vaccine is safe and effective? | 1. Yes 2. No |
| Do you think being vaccinated for HPV reduces the risk of having an HPV infection? | 1. Yes 2. No |
| Do you think the HPV vaccine will not lead to complicated sexual activities? | 1. Yes 2. No |
| Do you think vaccinating your daughter against HPV will not encourage them to start sexual activity ? | 1. Yes 2. No |
| Do you think the HPV vaccine promotes risky sexual behaviors among teenagers? | 1. Yes 2. No |
| Would you like to vaccinate your daughter against HPV if the vaccination is freely available? | 1. Yes 2. No |
| Do you think to decide whether your children should be vaccinated against HPV, information on HPV helps you? | 1. Yes 2. No |
| Are you afraid of mild side effects of the HPV vaccine for your daughter? | 1. Yes 2. No |
| Do you fear infertility from the HPV vaccine for your daughter in the future? | 1. Yes 2. No |
| Do you think the HPV vaccine is effective in preventing cervical cancer? | 1. Yes 2. No |
| Do you think only those who are promiscuous would benefit from the vaccine? | 1. Yes 2. No |
